# Burden of malaria in children under five and caregivers’ health-seeking behaviour for malaria-related symptoms in artisanal mining communities in Ghana

**DOI:** 10.1186/s13071-021-04919-8

**Published:** 2021-08-21

**Authors:** Francois Dao, Sampson Kafui Djonor, Christian Teye-Muno Ayin, George Asumah Adu, Bismark Sarfo, Pricillia Nortey, Kwadwo Owusu Akuffo, Anthony Danso-Appiah

**Affiliations:** 1 Department of Epidemiology and Disease Control, School of Public Health, University of Ghana, Legon, Ghana; 2 Malaria Research and Training Center, Department of Epidemiology and Infectious Diseases, University of Science Techniques and Technologies of Bamako, Bamako, Mali; 3 National Malaria Control Programme, Accra, Ghana; 4 Department of Optometry and Visual Science, College of Science, Kwame Nkrumah University of Science and Technology, Kumasi, Ghana; 5 University of Ghana Centre for Evidence Synthesis and Policy, School of Public Health, University of Ghana, Legon, Ghana

**Keywords:** Prevalence, Malaria transmission, Vector control, Galamsey activities, Anaemia, *Plasmodium falciparum*, Childhood malaria, Caregivers’ behaviour, East Akim, Ghana

## Abstract

**Background:**

Artisanal mining creates enabling breeding ground for the vector of malaria parasites. There is paucity of data on the effects of artisanal mining on malaria. This study assessed burden of malaria and caregivers’ health-seeking behaviour for children under five in artisanal mining communities in East Akim District in Ghana.

**Methods:**

A cross-sectional study involving caregivers and their children under five was conducted in three artisanal mining communities in the East Akim District in Ghana. Caregivers were interviewed using a structured questionnaire. Finger prick blood samples were collected and analysed for haemoglobin concentration using a rapid diagnostic test, and thick and thin blood smears were analysed to confirm the presence of malaria parasites.

**Results:**

Of the 372 children under 5 years included in the study, 197 (53.1%) were male, with a mean age (± SD) of 23.0 ± 12.7 months. The proportion of children with malaria (*Plasmodium falciparum* and *P. malariae*) was 98.1% and 1.9%, respectively, whilst the proportion with anaemia (Hb < 11.0 g/dl) was 39.5% (*n* = 147). Almost all caregivers were female (98.9%), and 28.6% (*n* = 106) did not have access to any malaria control information. Caregivers associated malaria infection with mosquito bites (68.3%, *n* = 254) and poor sanitation (21.2%, *n* = 79). Malaria in children under five was significantly associated with anaemia (OR 11.07, 95% CI 6.59–18.68, *n* = 111/160, 69.4%; *P* < 0.0001), residing close to stagnant water (≤ 25 m) from an artisanal mining site (AOR 2.91, 95% CI 1.47–5.76, *P* = 0.002) and caregiver age younger than 30 years (OR 0.44, 95% CI 0.208–0.917, *n* = 162, 43.55%, *P* = 0.001).

**Conclusions:**

There is a high burden of malaria and anaemia among children under five in artisanal mining communities of the East Akim District, and far higher than in non-artisanal mining sites. Interventions are needed to effectively regulate mining activities in these communities, and strengthen malaria control and health education campaigns to curtail the high malaria burden and improve health-seeking behaviour.

**Graphical abstract:**

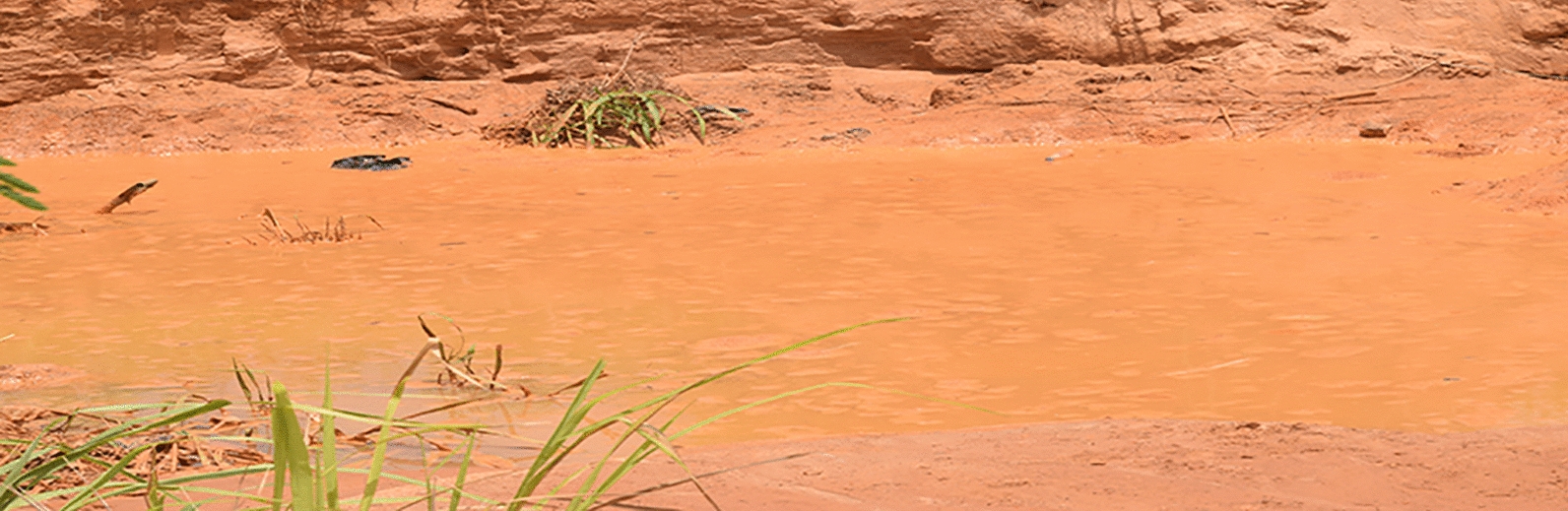

**Supplementary Information:**

The online version contains supplementary material available at 10.1186/s13071-021-04919-8.

## Background

After decades of control efforts, malaria still poses a serious public health threat, with 229 million estimated reported cases in 2019 [1] and 405,000 attributable deaths, of which two-thirds (272,000) occurred in children under 5 years of age [2]. Deaths from malaria are caused mainly by the acute form of the disease, but much more is due to subtle effects, including anaemia. Anaemia remains a major complication and risk of death from malaria. In 2018, it was estimated that up to 79% of children under five in high-burden areas in Africa who were diagnosed with malaria had anaemia [3]. Malaria is the number one cause of morbidity, accounting for about 38% of all outpatient illnesses, and about 31% of all deaths in children under five [4], with almost all cases (97%) caused by *Plasmodium falciparum* [5]. According to the World Health Organization (WHO), the African Region accounted for 94% of all malaria deaths in 2018, and despite the relatively lower number of 180,000 deaths in that year, the region was still responsible for 85% of the deaths recorded [2].

A systematic review assessing patterns of malaria variation by age with respect to severity, transmission intensity and seasonality in sub-Saharan Africa found clinical malaria burden to be higher in younger age groups. Hospital admissions were also higher among younger children, with higher levels of mortality among infants [6].

In Ghana, malaria accounted for 10.4 million of outpatient department (OPD) visits in 2016 and was responsible for a case fatality rate of 0.32 among children under five [7]. That same year (2016), the East Akim District of the Eastern Region of Ghana, which is well known for its mining activities, including artisanal mining, recorded prevalence of 34.1% with the malaria rapid diagnostic test (m-RDT) among children aged 6 to 59 months, the highest in the region [8]. Several studies have sought to link the activities of artisanal mining of gold with the increased prevalence of malaria [9–11]. Deforestation, unrehabilitated mining pits containing stagnant water bodies and other mining-related activities are known to promote the proliferation of the female anopheles mosquitoes, the vector for the *Plasmodium* parasite responsible for most of the malaria cases in Ghana. Lack of adequate housing, with unsealed windows and doors allowing free entry of mosquitoes, and living less than 25 m from a stagnant water body have also been found to be factors associated with increased exposure to malaria risk [11, 12].

However, there is a paucity of data on the burden of malaria in children under five in the East Akim District. Furthermore, the extent to which ongoing artisanal mining is impacting the burden of malaria is largely unknown. Therefore, this study sought to assess the burden of malaria in children under five as well as caregivers’ knowledge and healthcare-seeking behaviour for malaria-related symptoms in their children in an artisanal mining community in Ghana. Findings from this study could be utilized in planning future malaria control activities in similar settings.

## Methods

### Type of study design and study area

**Fig. 1:**
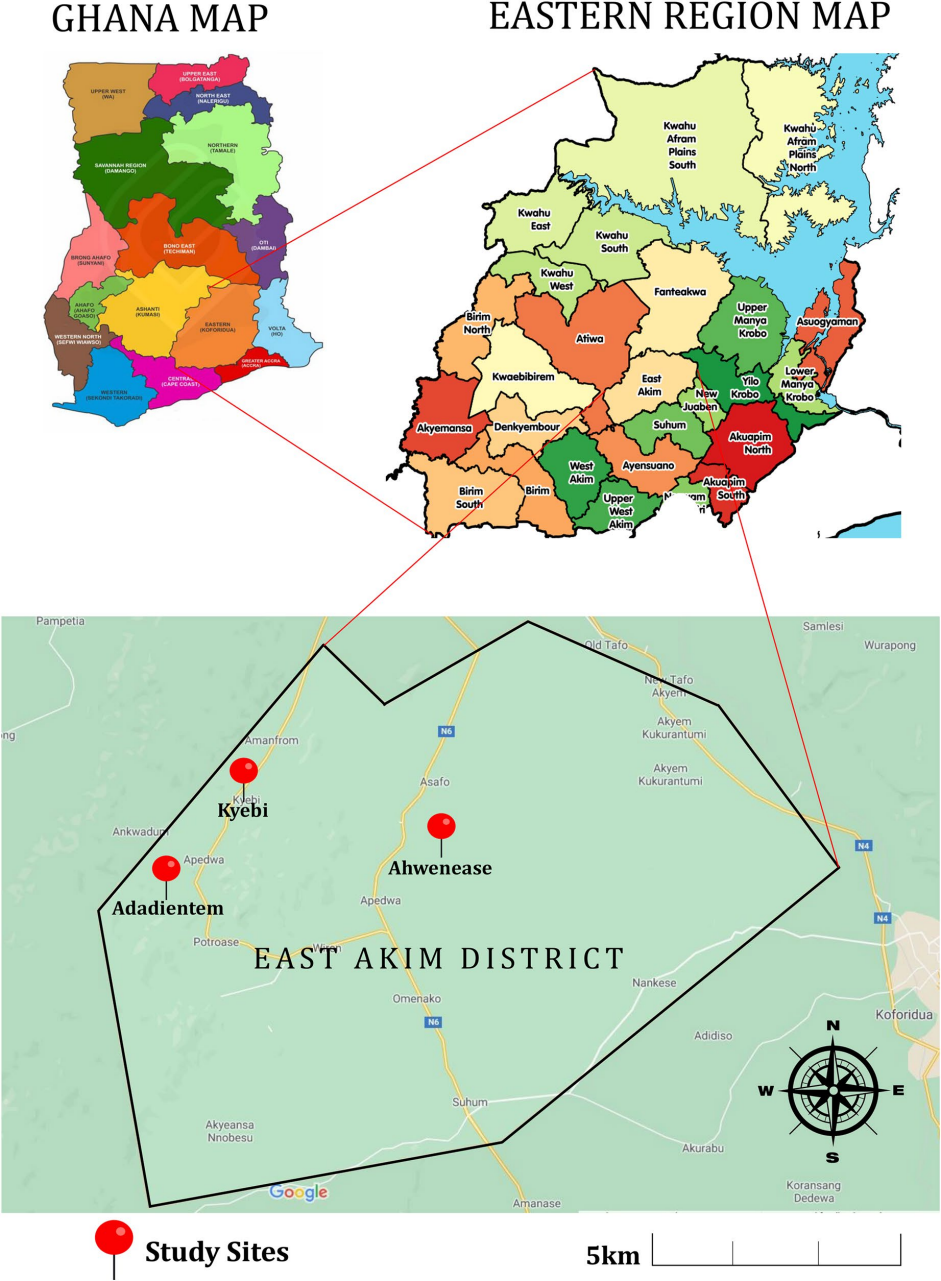
District map of East Akim

The study used a cross-sectional design and employed quantitative methods. It was conducted in three localities (Kyebi, Adadientem and Ahwenease) of East Akim District, an artisanal mining area in the Eastern Region of Ghana (Fig. 1). The East Akim Municipal Assembly has a total area of about 725 km^2^. According to the 2010 Population and Housing Census [13], the population of the Akim Municipality was estimated at 167,896 inhabitants. The municipality is located in the western semi-equatorial zone, which is characterized by two main rainfall seasons, May–June and September–October, with average annual rainfall of between 125 and 175 mm. Temperatures are relatively uniform, ranging from 26 °C in August to 30 °C in March. The relative humidity is generally high year-round, ranging between 70 and 80%. The municipality is in the semi-deciduous rainforest with a forest reserve covering about 108.8 km^2^, representing approximately 15% of the total area. The district is heavily endowed with mineral deposits—gold, diamond, bauxite, etc. But the mining sector is dominated by the activities of artisanal miners, which exist in almost every community of the district. The district is made up of 27 communities, of which 21 are rural.

The municipality is divided into seven health administrative sub-municipalities: Amanfrom, Apedwa, Asafo, Asiakwa, Bunso, Kibi and Maase. There is one municipal hospital, one polyclinic, four health centres, 15 community-based health planning service (CHPS) compounds and 29 demarcated CHPS zones. Malaria, upper respiratory infections, rheumatism and joint pain, intestinal worm infestation and acute respiratory tract infection are the typical cases reported to health facilities. With the introduction of the National Health Insurance Scheme (NHIS) in 2006, services and care rendered by these facilities are covered by the scheme, although the roughly 30% of patients not registered with the NHIS would have to pay out-of-pocket for services received.

### Study population

The study population comprised children under 5 years of age and their caregivers who had lived in the East Akim artisanal mining communities for at least a month. One month was chosen to reflect the incubation period of the *Plasmodium* parasites [12]. Children who were eligible to be included but whose caregivers failed to give consent to the study protocols were excluded. Each child was linked directly to their caregiver in the study.

### Sample size determination and sampling

According to the Ghana Malaria Indicator Survey 2016 [8], the Eastern Region recorded the highest malaria cases by microscopy, with 31.1% in children aged 6–59 months (under 5 years). Using this proportion, the minimum sample size was derived using Cochran's formula:

$$N = \frac{{z^{2} \left( {p \times q} \right)}}{{d^{2} }},$$

where *N* = sample size; *p* = malaria proportion by microscopy in the Eastern region (31.1%); *q* = 1 − *p*; *z* = the critical probability value for a confidence level of 95% (1.96) and *d* = 5% margin of error (0.05).

This gives $$N = \frac{{\left( {1.96} \right)^{2} \left( {0.311 \times 0.689} \right)}}{{\left( {0.05} \right)^{2} }} = 329.27.$$

With an assumed estimate of 10% non-response rate, a minimum total sample size of 362 children (one child per caregiver) was obtained for the entire study.

**Fig. 2:**
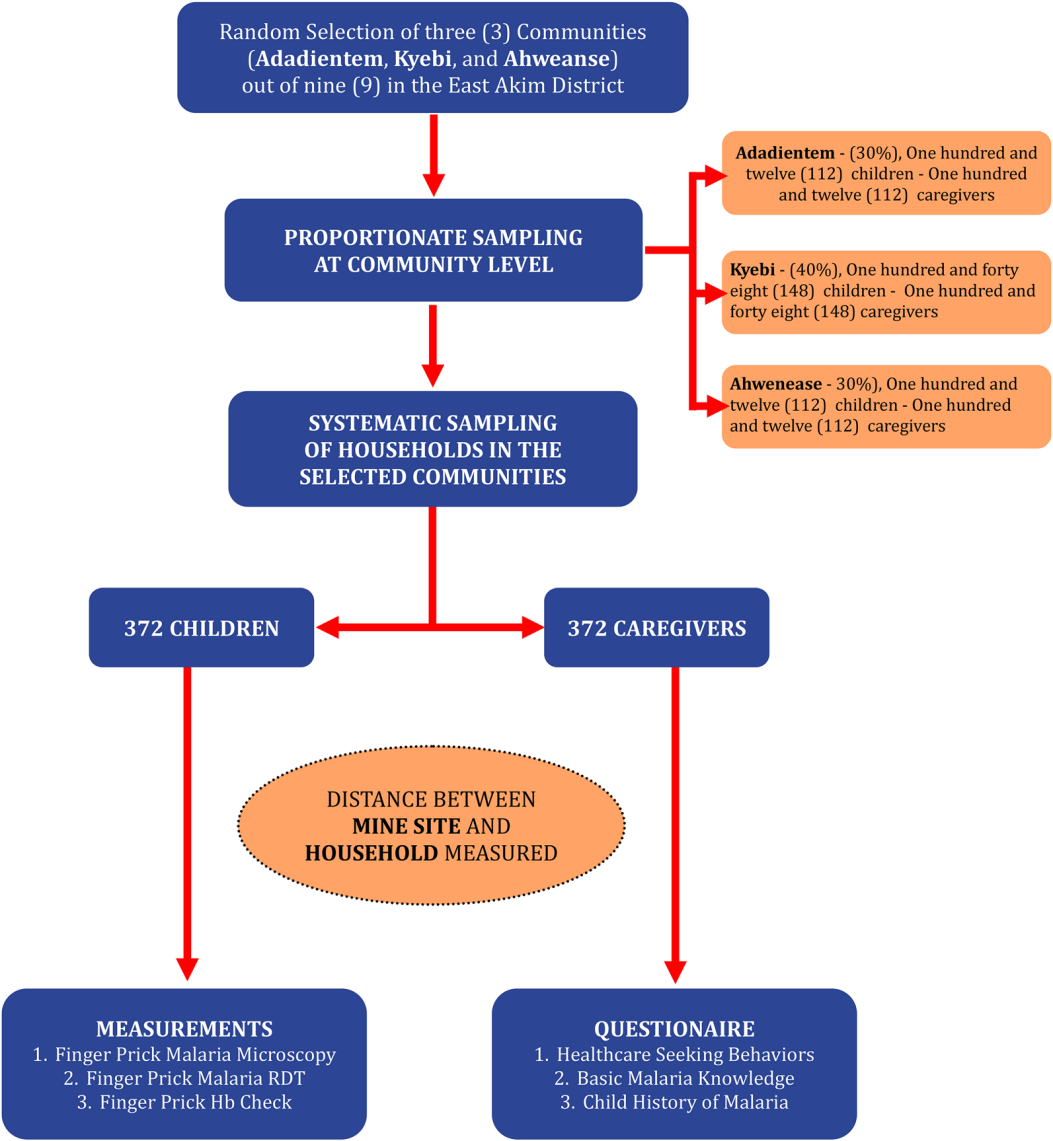
Flow chart illustrating the recruitment procedure

The East Akim District has nine artisanal mining communities with different population sizes. Three communities were randomly selected for this study, namely Adadientem, Kyebi and Ahwenease. The communities were sampled proportionately based on the community size and translated into a ratio of 30:40:30, respectively. Figure 2 summarizes the recruitment process.

A total of 112 children under 5 years from Adadientem, 148 from Kyebi and 112 from Ahwenease, together with their caregivers, were sampled for the study. With the knowledge of estimated total number of houses in these communities, a proportionate sampling method was used to select the number of houses included in the study. The researcher tossed a pen, and where the tip of the pen pointed marked the direction of movement. The first house in that direction was chosen, and the third number from that house was selected in that order. One child under 5 years was selected in each house; in houses with more than one qualified participant, a simple random sampling was used to select one of them. Also, an adjacent house with a qualified participant was used to replace houses without qualified participants or with a participant who declined to participate in the study. The distance between a house and a mining site with stagnant water was determined using a Global Positioning System receiver (Garmin eTrex 20).

### Blood sample collection technique

Finger prick blood samples were collected from children under five to measure the haemoglobin concentration using the HemoCue^®^ Hb 201+ system and to prepare thick and thin smears for observation under a light microscope (Olympus CX22 binocular microscope, 100 × 100) for identification of asexual malaria parasites after staining with 10% Giemsa. According to standard WHO procedures, haemoglobin concentrations and blood smear results were then reported as soon as possible after observation by certified microscopists. Malaria parasitaemia was defined as the presence of an asexual form of any *Plasmodium* species detected by microscopy. Asymptomatic malaria was defined as the presence of an asexual form of any microscopically detected plasmodial species with the absence of symptomatic malaria or history of fever over the last 2 weeks. Participants were also examined with the SD BIOLINE Malaria Ag P.f rapid diagnostic test (RDT; HRP2/pLDH). Participants who tested positive with RDT or microscopy were referred for malaria treatment at the nearest health centre.

### Questionnaire and data collection technique

The questionnaire used in this study had four main sections. The first section collected information on children's and caregivers’ demographic characteristics; the second, data from caregivers on their knowledge of malaria transmission, signs and symptoms; the third, information on their child’s health and history of malaria signs and symptoms; and the last on caregivers’ health-seeking behaviour.

### Quality assurance and quality control

The survey was conducted by experienced research assistants who were trained in the study protocols. All samples taken were marked with a participant identification number and recorded in a register. Two certified microscopists, who were blinded to the RDT results, carried out the microscopy for malaria parasites. A third microscopist was consulted in the case of any divergence greater than or equal to 50%. The HemoCue system and microscopes were calibrated and verified prior to use in the study. The RDT kits were approved by the Food and Drugs Authority (FDA), as recommended by the Ghana National Malaria Control Programme (NMCP). The questionnaire was completed and verified by the data collection team for accuracy, consistency and completeness.

### Data processing and analysis

A mobile app (REDCap^®^ software) was downloaded as a Microsoft Excel file and used for the data collection. Stata version 15.0 was used for the statistical analysis. The level of malaria parasitaemia was determined by counting the number of asexual parasites in blood film against 200 leucocytes counted alongside these parasites, and by assuming 8000 leukocytes/ml; the ratio obtained was then multiplied by the leucocyte count to determine the parasite density per microlitre of blood for each participant with a positive malaria test. Haemoglobin values less than 11.0 g/dl were classified as anaemia present, and values above 11.0 g/dl described as no anaemia. The categorical variables were presented as frequencies and percentages. Univariate logistic regression was run for all the variables, and those found to be statistically significant at *P* < 0.05 were further analysed using multivariate logistic regression. All estimates were presented with their 95% CIs.

## Results

### Socio-demographic characteristics of children under five

Though we set out to recruit a minimum of 362 children, the study sampled a total of 372 children, with mean age of 23 months (SD 12.7 months). Two thirds (66.9%) of the children were aged ≤ 11 months and 197 (53.1%) were male.

### History of presence of malaria signs/symptoms and intensity among the children

Two hundred and seventy-five (275) children reported an episode of fever more than 1 month prior to the study. The fever was of moderate intensity in 208 (75.6%) children, with 197 (71.6%) of the children who had fever receiving medical care from the hospital or clinic. Among those not treated in either the hospital or clinic, 34 (12.4%) caregivers believed that self-medication was a better option. Regarding the history of the presence of malaria signs and symptoms and intensity, 19 (5.1%), 11 (2.9%) and 20 (5.4%) children experienced convulsions, pallor and somnolence, respectively. The number of reported symptoms increased according to the duration of the disease: between 2 weeks and 1 month for convulsions (16; 84.2%), pallor (6; 54.6%) and somnolence (16; 80.0%). Symptom intensity was more severe with convulsions (17; 89.5%) and somnolence (17; 85.0%) than with pallor (5; 45.5%). A significant proportion of cases of convulsions, pallor and somnolence were reported to have been treated in a health facility [16 (84.2%), 8 (72.7%) and 17 (85.0%), respectively] (Additional file 1: Table S1).

### Children’s medical history

In this study, 134 (36.0%) children were reportedly experiencing a malaria-related sign or symptom on the day of the survey. Of the 156 (41.9%) children who were reported to have been sick with malaria in the past month, treatment was sought by caregivers for 143 (91.7%), mostly in a health facility (109; 76.2%). Twenty-four (16.8%) visited a pharmacy, one (0.7%) caregiver engaged in self-medication, and two (1.4%) sought care from a faith healer. Sixty (37.0%) children were asymptomatic carriers of malaria.

### Socio-demographic characteristics of caregivers

A total of 372 caregivers were interviewed, of whom the majority (368; 98.9%) were female, with a mean age of 26.5 years (SD 5.4), and a majority were married (67.1%). Most caregivers had a low level of education, with primary or junior secondary school (JSS)/senior high school (SHS) representing 75.8%. One hundred and fifty-eight (42.5%) were self-employed, and the vast majority (365; 98.1%) were of the Christian faith (Table 1).

**Table 1:** Socio-demographic characteristics of caregivers

Characteristic	Frequency (*n* = 372)	Percentage
Sex of caregiver		
Male	4	1.1
Female	368	98.9
Educational status of caregiver		
No formal education	24	6.5
Primary/JHS/SHS	282	75.8
Secondary	58	15.6
Tertiary (university)	8	2.1
Occupation (caregiver)		
Civil servants	12	3.2
Self-employed	158	42.5
Trading	87	23.4
Unemployed	91	24.5
Others	24	6.4
Religion (caregiver)		
Christianity	365	98.1
Islam	4	1.1
Traditional (fetishism)	2	0.5
Other	1	0.3
Marital status (caregiver)		
Single	113	30.5
Married	249	67.1
Divorced	3	0.8
Separated	6	1.6

*JHS* junior high school, *SHS* senior high school

### Socio-environmental factors

The majority of caregivers (349; 93.8%) said that they had well-sealed windows and doors, with 304 (81.7%) having well-screened windows. Two hundred and seventy-three respondents (42.5%) had a place of residence close to a mine site (within 25 m from the mine site), and 198 (53.2%) had stagnant and open wells created from mining activities close to their place of residence.

### Caregivers’ health-seeking behaviour

Regarding caregivers’ health-seeking behaviour, 234 (62.9%) caregivers sought medical care for their children at health facilities. Forty-seven (12.6%) engaged in other practices such as self-medication or visiting a spiritualist or herbalist. Most (79.4%) caregivers who resorted to other forms of treatment rather than seeking care from health facilities said they practiced self-medication. Almost 9% (8.8%) of those who sought other forms of care said they did so because of high healthcare cost.

### Caregivers’ awareness and knowledge of malaria

The results showed that 106 (28.6%) caregivers did not have access to any malaria control information. Thirty-four (9.1%) accessed malaria information at least six times per month. One hundred and twenty-seven (40.8%) of the respondents accessed malaria information through the radio, whilst 102 (40.8%) accessed through television. Few respondents accessed malaria information from health workers and friends. Approximately 350 (94%) caregivers knew about malaria and were aware of how it is transmitted, with most associating it with mosquito bites (254; 68.3%) and poor sanitation (79; 21.2%).

### Association between malaria and anaemia among the children

Two hundred and two (54.3%) of the children tested positive for malaria by malaria RDT, whilst 160 (43.0%) of the slides showed the presence of malaria parasite by microscopy, indicating an 11% difference between the two diagnostic criteria. *Plasmodium falciparum* constituted the majority of the infections (98.1%). One child had mixed infections of *P. malariae* and *P. falciparum.* The mean Hb of the children was found to be 11.4 (95% CI 11.2–11.5) g/dl, with a minimum Hb reading of 7.2 g/dl and maximum of 15.2 g/dl. One hundred and forty-seven (39.5%) of the children had anaemia (Hb < 11.0 g/dl) (Additional file 1: Table S2).

Using the thick/thin blood smear, which is the gold standard for malaria diagnosis, the prevalence of malaria-associated anaemia in children under five was found to be 69.4%. Anaemia was found to be associated with malaria in children under five (Table 2). Among all 147 children who had anaemia, 136 (92.5%) tested positive for malaria by RDT (OR 29.8, 95% CI 15.1–58.7, *P* < 0.0001) whilst 111 (75.5%) also tested positive for malaria from blood smear (OR 11.07, 95% CI 6.8–18.1, *P* < 0.0001).

**Table 2:** Association between anaemia and malaria among children under 5 years living in artisanal mining communities in Ghana

Variables	Anaemia status		Crude OR (95% CI)
	Absent (%)	Present (%)	
Malaria (RDT results)			
Negative	159 (70.7)	11 (7.5)	0.07
Positive	66 (29.3)	136 (92.5)	29.8 (15.1–58.7)
Malaria (smear results)			
No MPs seen	176 (78.2)	36 (24.5)	0.20
MPs seen	49 (21.8)	111 (75.5)	11.1 (6.8–18.1)
*Plasmodium* infection			
Negative	176 (78.2)	36 (24.5)	0.20
Mono-infection (asexual form)	49 (21.8)	110 (74.8)	11.0 (6.7–18.0)
Mixed infection (asexual form)	0 (0.0)	1 (0.3)	–

*RDT* rapid diagnostic test, *CI* confidence interval, *OR* odds ratio, *MPs* malaria parasites

From bivariate analysis of caregivers’ socio-demographics and malaria infection, significant associations with infection were found for both child and caregiver age (Additional file 1: Table S2). Caregivers who were 30 years old and older were less likely to have their children infected with malaria compared to those younger than 30 (OR 0.4, 95% CI 0.2–0.9, *P* = 0.017). However, caregivers’ marital status (*P* = 0.753), employment (*P* = 0.928) and formal educational status (*P* = 0.753) and the sex of the child (*P* = 0.206) were all found not to be significant.

### Caregiver’s knowledge of malaria and incidence of malaria in children under five from bivariate analysis

The bivariate analysis revealed that knowledge of malaria control information among caregivers was protective against malaria infections in children (OR 0.7, 95% CI 0.4–1.3, *P* = 0.207). However, the difference between good and poor knowledge of malaria control information among caregivers was not statistically significant in influencing malaria infections in children under five.

### Bivariate analysis of socio-environmental factors and malaria infection

The study found a significant association between place of residence close to mine sites and malaria infection. Children of caregivers who lived less than or equal to 25 m from mine sites were 4.63 times as likely to be infected with malaria as those who lived more than 25 m from mine sites (OR 4.6, 95% CI 2.6–8.5, *P* = 0.001). Furthermore, children whose caregivers lived 25 m or less from stagnant clean water wells due to mining were 2.67 times as likely to be infected with malaria as those who did not live close to mine sites (OR 2.8, 95% CI 1.7–4.2, *P* = 0.001). However, the presence or absence of window door screens did not show any statistical significance with regard to malaria infection (*P* = 0.916).

### Multiple logistic regression analysis

All variables described in Additional file 1: Table S2 were examined for malaria infections in multivariate analysis. These variables included caregiver and child age, caregiver marital status, employment, education, frequency with which child contracted malaria, closeness (≤ 25 m) of residence to mining-created stagnant wells, closeness of residence to mining site, and caregiver’s access to malaria control information. These were variables that were found significant at the bivariate analysis level or considered to have a theoretically significant influence on malaria infection in artisanal and small-scale mining (ASM) communities. Only the frequency with which a child contracted malaria and the closeness (≤ 25 m) of the residence to mining-created stagnant wells remained significant in the multivariate model. Caregivers whose homes were close (≤ 25 m) to stagnant water wells due to mining were 2.9 times as likely to have their child infected with malaria as those who did not live close to stagnant clean water wells (OR 2.9, 95% CI 1.5–5.8, *P* = 0.002). Children who often contracted malaria were also twice as likely to have malaria infections as those who did not often contract malaria (OR 2.0, 95% CI 1.1–3.8, *P* = 0.023). No other factors were found to be significantly associated with malaria infections in ASM sites in the multivariate analysis.

## Discussion

### Malaria among children under 5 years in ASM sites in Ghana

The study assessed the malaria burden among children under five in the artisanal mining communities of East Akim and caregivers’ knowledge in relation to malaria. The study found 54.3% cases of malaria using RDT and 43% with microscopy. Malaria was a good predictor of anaemia, with malaria-associated anaemia found in 69.4% of cases. Caregivers seeking medical care from health facilities accounted for 62.9%, of whom 94% had knowledge of malaria and mode of transmission of the infection. Living in a residence less than 25 m from a stagnant clear water well due to artisanal mining increased the risk of malaria for children under five by threefold.

Several studies have sought to estimate the burden of malaria in children under five, usually with wide variations in prevalence. A survey by Yankson et al., which sought to assess the risk of malaria in children under five by analysing the 2016 Ghana Demographic Health Survey data, found malaria prevalence of 22.1% [14]. An intervention study in children under five in Lagos, Nigeria, showed malaria parasitaemia in 58.8% at baseline [15]. Another study in Uganda found malaria prevalence of 19.0% and a study in rural Malawi found 33.8% prevalence using a cross-sectional survey [16, 17]. Generally, high malaria prevalence in children under five has been associated with increasing child age, low educational status of mother, and coming from both a poor household and rural area [14, 16–18]. In this study, at artisanal mining areas, malaria prevalence in children under five was relatively high (43%) compared to previously cited studies. The high malaria prevalence rate in artisanal mining areas could be due to massive environmental changes that affect vector abundance and transmission behaviour [19]. ASM activity leaves stagnant dirty water that serves as a breeding ground for mosquitoes and promotes the spread of malaria. We also found that having frequent episodes of malaria was significantly associated with malaria infection in these artisanal mining communities. Despite our inability to identify a cause-and-effect relationship in this cross-sectional study, we strongly believe that ASM and its associated environmental conditions predispose children under 5 years to malaria infection, and are reasons why children in these study sites have frequent episodes of malaria. Until breeding grounds of mosquitoes left behind by ASM are identified and controlled, infection will continue. This explains why malaria could be frequent in these children.

### Malaria-associated anaemia among children under 5 years in ASM sites

The prevalence of malaria-associated anaemia was 69.4% in this study. A child under five with a positive malaria RDT had 30-fold increased odds of anaemia compared to a similar child with negative malaria RDT. Likewise, having a positive malaria smear increased the odds of a child under five having anaemia by 11-fold. Similar results have been reported in studies in low-resource mining settings of Okada in Nigeria, the Bonikro mining area in central Cote d'Ivoire, and Luangwa District in Zambia [20–22]. Erythrocyte lysis and phagocytosis and sequestration of parasitized red blood cells have been proposed as possible explanations for malarial anaemia, with recent data also indicating bone marrow suppression and ineffective erythropoiesis as possible pathways [23, 24]. A large study in Ghana involving 2123 children conducted around the same period as the current study found anaemia and malaria prevalence of 35.6% and 20.3%, respectively, in children under 5 years [25]. An earlier household survey of 7739 children under 5 years in 2014 also found 40% anaemia prevalence [26]. About 3 years later, where several conditions contributing to anaemia in the country should have been better, anaemia prevalence of over 60% would not have been expected—and is strongly associated with malaria in this study and owing to artisanal mining in these sites. Mining activities always leave behind environmentally unfavourable conditions such as abundant bushy areas and dug holes that accumulate dirty stagnant water that serves as breeding grounds for mosquitoes and the spread of malaria.

### Asymptomatic and *Plasmodium* species-specific malaria in ASM sites in Ghana

This study saw about a third of asymptomatic carriers of malaria parasites among children under five. Similar findings have been reported in several studies, including artisanal gold mining settings in Guyana. Inadequate malaria treatment, which limits parasite densities without actually eliminating them, has been linked to clinically silent malaria. Asymptomatic carriers serve as reservoirs for malaria parasites, likely becoming a channel for intense malaria transmission when mosquitoes start breeding during the rainy season [19, 27–29].

About half of the children tested positive for malaria by RDT, whilst a slightly lower proportion (43.0%) had positive slides for malaria in this study. *Plasmodium falciparum* accounted for the majority of malaria infections, consistent with the World Health Organization report, which indicates that *P. falciparum* remains the most common of the five *Plasmodium* species that are known to cause malaria disease among Africans [2]. Additionally, findings support the data from the Ghana Health Service (GHS), where malaria caused by *P. falciparum* species accounts for almost 97% of cases [4, 5].

### Effect of ASM on malaria infections in nearby communities

In the current study, among the factors studied that could influence the risk of malaria infection, children under five whose caregivers lived less than 25 m from an open stagnant clean water well as a result of mining had significantly increased risk of malaria infection. This finding is consistent with studies conducted in both Ghana and southern Ethiopia, which reported children under five living near mining areas being disproportionately exposed to mosquitoes bites [30, 31]. Malaria and its associated anaemia can lead to significant morbidity and mortality. Several studies [14, 16–19] have pointed out how the high rate of malaria infection and its associated anaemia as evidenced in the current study could be a result of ASM in the study community, which requires expedited action to curtail the situation. Although our study concentrated only on malaria, ASM is also associated with several other health implications ranging from nutritional disorders to respiratory issues, renal diseases, poisoning, cardiovascular and gastrointestinal diseases, tuberculosis and pneumonia, among others [32]. Artisanal mining has been a cancer in most African countries, polluting water bodies and causing various disease conditions and other social problems, and the earlier that governmental and non-governmental institutions stand up against it, the better for the health and well-being of all.

### Other factors influencing malaria infection in ASM sites

The results of this study revealed that children whose caregivers had good knowledge of malaria transmission and prevention had 30% less risk of malaria infection than those with no information or poor knowledge. Some studies have attributed caregivers’ educational level to children's risk of malaria infections, with children whose caregivers had no education at all having higher risk than those with higher education [14]. It could be assumed that caregivers with higher formal education are more likely to have greater awareness and knowledge of malaria infection, as found in the study by Mukomena et al. in Dembele kebele in south-eastern Ethiopia [33]. Our study, however, found that caregivers’ educational level influenced malaria among their wards only at the bivariate level. Though it is unclear why this was not significant at the multivariate level, the generally low level of education throughout the ASM communities could be a factor.

The study also found that children under five with a history of frequent malaria had significantly greater odds of having malaria during the study period than those with no history of frequent malaria infection. This finding could be attributed to inadequate treatment of previous malaria infections by caregivers, resulting in children becoming asymptomatic carriers of infection. This assertion is further strengthened by the study findings, with a significant proportion of caregivers seeking malaria treatment from pharmacies, faith healers, spiritualists and herbalists or self-medicating rather than seeking care from a health centre. Previous studies by Nacher et al. [27] and Douine et al. [34] in French Guiana and the Amazon have shown similar findings.

Studies in Tanzania and Uganda found that older children were at greater risk of being infected with malaria compared to infants [35, 36]. This could be explained by the fact that infants have immunity acquired from their mothers, including passive transfer of antibodies through breastfeeding. With increasing age, this immunity starts to wane, and hence children are at increased risk of malaria infection before they begin to develop their own immunity following repeated infections [37, 38]. The present study, however, found no significant difference in malaria infections between children up to 11 months and those 12 months and older.

Despite caregiver knowledge and child history of malaria being significant factors in predicting malaria infection in this study, ASM remains the major source of exposure to infection and hence requires prudent measures to curb it.

### Limitations and strengths of the study

Our study has a number of limitations. The first is the likelihood that participants may have brought a malaria infection from a different location due to travel. To ensure that our study findings were representative of the East Akim District, participants were selected based on having lived in the district for at least a month, which corresponds to the most frequent malaria incubation period with *P. falciparum* and the longest with *P. malariae* as indicated by the Centers for Disease Control and Prevention (CDC) [12].

Another drawback of this study is the possibility of incorrect malaria diagnosis among participants, as correct diagnosis requires technical expertise and skill. To mitigate this risk, quality control checks were performed on RDT test kits, and their results were accepted only when the control bands on the kits supported the interpretation of either a positive or negative test result. Additionally, the manufacturer’s standard operating procedures were strongly adhered to. For malaria smear examinations, two certified and accredited microscopists who were blinded to the RDT results were engaged. A third microscopist was available in the case of divergent decisions in film/smear examination and reporting.

## Conclusions

This study reveals a high prevalence of malaria parasite and infection (54.3%) among children in artisanal mining communities of the East Akim District—a level much higher than that of the other districts, regions and the country at large. Not only is the prevalence high, but the asymptomatic (carrier) proportion is also alarming, with these infections being strongly associated with malaria. Stagnant residual water from mining activities in close proximity (≤ 25 m) to homes in these communities, inadequate control and experience of younger caregivers in handling the situation, and a child having a history of frequent malaria were factors associated with anaemia and malaria infection in this study. In light of the serious adverse effects associated with malaria among children, there is a pressing need to effectively regulate mining activities in resident communities, and to strengthen malaria control and education campaigns in these communities to reduce the high malaria prevalence and improve the health-seeking behaviour of community residents.

## Supplementary Information

**Additional file 1: Table S1.** History of malaria-related signs/symptoms and caregivers’ health-seeking behaviour for their child’s symptoms including what action they took, whether they visited the health facility and the reasons for not visiting a health facility. Figures represent number and percentage (in parentheses). **Table S2.** Odds ratio of factors that influence malaria infections in children under five in East Akim district, Ghana.

## Data Availability

The datasets used and/or analysed during the current study are available from the corresponding author on reasonable request.

## Abbreviations

AOR: Adjusted odds ratio
CHPS: Community-based health planning services
CI: Confidence intervals
COR: Crude odds ratio
FDA: Food and Drugs Authority
GHS: Ghana Health Service
m-RDT: Malaria rapid diagnostic test
NHIS: National Health Insurance Scheme
OPD: Outpatient department
OR: Odds ratio
RDT: Rapid diagnostic test
SD: Standard deviation
SHS: Senior high school
WHO: World Health Organization
