# Supplementary material for: Burden of malaria in children under five and caregivers’ health-seeking behaviour for malaria-related symptoms in artisanal mining communities in Ghana
Source: Parasit Vectors. 2021 Aug 21;14:418. doi: 10.1186/s13071-021-04919-8 (PMC8380373; doi:10.1186/s13071-021-04919-8)
Supplement: Supplementary file 1 — Additional file 1: Table S1. History of malaria-related signs/symptoms and caregivers’ health-seeking behaviour for their child’s symptoms including what action they took, whether they visited the health facility and the reasons for not visiting a health facility. Figures represent number and percentage (in parentheses). Table S2. Odds ratio of factors that influence malaria infections in children under five in East Akim district, Ghana. [file 13071_2021_4919_MOESM1_ESM.docx]

Additional file 1: Table S1. History of malaria-related signs/symptoms and caregivers’ health seeking behaviour for their child’s symptoms including what action they took, whether they visited the health facility and the reasons for not visiting a health facility. Figures represent number and percentage (in parentheses).

| Sign and symptoms ^a^ | Presence | | | | Intensity | | | What has been done? | | | | Why not Health facility? | | | |
| --- | --- | --- | --- | --- | --- | --- | --- | --- | --- | --- | --- | --- | --- | --- | --- |
|  | Yes, now | 1 day-1 week | 2 week-1 month | > 1-month | Mild | Moderate | Severe | Hospital | Traditional medicine | Self-medication | Spiritualist | Health facility care expensive | Didn’t have money | Health facility care not effective | Self-medication better |
| Fever (*n* = 275) | 24 (8.7) | 82 (29.8) | 125 (45.5) | 44 (16.0) | 9 (3.3) | 208 (75.6) | 58 (21.1) | 197 (71.6) | 27 (9.8) | 51 (18.6) | 0 | 21 (7.6) | 7 (2.6) | 16 (5.8) | 34 (12.4) |
| Chills (*n* = 164) | 7 (4.3) | 37 (22.6) | 98 (59.8) | 22 (13.4) | 3 (1.8) | 129 (78.7) | 32 (19.5) | 132 (79.9) | 23 (14.0) | 10 (6.1) | 0 | 9 (5.5) | 6 (3.7) | 8 (4.9) | 10 (6.1) |
| Headaches (*n* = 112) | 5 (4.5) | 28 (25.0) | 64 (57.1) | 15 (13.4) | 0 | 103 (92.0) | 9 (8.0) | 84 (75.0) | 19 (17.0) | 9 (8.0) | 0 | 18 (16.1) | 0 | 1 (0.9) | 9 (8.0) |
| Abdominal pain (*n* = 114) | 16 (14.0) | 13 (11.4) | 69 (60.5) | 16 (14.0) | 0 | 96 (84.2) | 18 (15.8) | 80 (70.2) | 25 (21.9) | 9 (7.9) | 0 | 7 (6.1) | 1 (0.9) | 2 (1.8) | 24 (21.1) |
| Vomiting (*n* = 119) | 5 (4.2) | 14 (11.8) | 82 (68.9) | 18 (15.1) | 21 (17.7) | 82 (68.9) | 16 (13.5) | 114 (95.8) | 4 (3.4) | 0 | 1 (0.8) | 0 | 5 (4.2) | 0 | 0 |
| Loss appetite (*n* = 119) | 11 (9.2) | 18 (15.1) | 77 (64.7) | 13 (10.9) | 5 (4.2) | 98 (82.4) | 16 (13.5) | 109 (91.6) | 10 (8.4) | 0 | 0 | 6 (5.0) | 3 (2.5) | 0 | 1 (0.8) |
| Convulsions (*n* = 19) | 0 | 0 | 16 (84.2) | 3 (15.8) | 0 | 2 (10.5) | 17 (89.5) | 16 (84.2) | 0 | 0 | 3 (15.8) | 1 (5.3) | 2 (10.5) | 0 | 0 |
| Pallor (*n* = 11) | 3 (27.3) | 1 (9.1) | 6 (54.6) | 1 (9.1) | 0 | 6 (54.6) | 5 (45.5) | 8 (72.7) | 3 (27.3) | 0 | 0 | 1 (9.1) | 1 (9.1) | 0 | 1 (9.1) |
| Drowsiness (*n* = 20) | 0 | 1 (5.0) | 16 (80.0) | 3 (15.0) | 0 | 3 (15.0) | 17 (85.0) | 17 (85.0) | 0 | 0 | 3 (15.0) | 0 | 0 | 3 (15.0) | 0 |

^a^ The signs and symptoms presented are independent of the presence of others. The total of all signs and symptoms should not be expected to sum up to 100%.

**Additional file 1: Table S2.** Odds ratio of factors that influence malaria infections in children under five in East Akim district, Ghana

| **Characteristics** | **Malaria infection** | | **Crude odds ratio (95% CI)** | ***P*-value** | **Adjusted odds ratio (95% CI)** | ***P*-value** |
| --- | --- | --- | --- | --- | --- | --- |
|  | Yes | No |  |  |  |  |
| Sociodemographic |  |  |  |  |  |  |
| Caregivers’ age |  |  |  | 0.017* |  |  |
| < 30years | 138 | 24 | 0.4 (0.2-0.9) |  |  |  |
| ≥30years | 195 | 15 | 1 |  |  |  |
| Educational status |  |  |  | 0.453 |  |  |
| ≥SHS | 26 | 40 | 0.8 (0.5-1.4) |  |  |  |
| SHS | 136 | 170 | 1 |  |  |  |
| Marital status |  |  |  | 0.753 |  |  |
| Married | 117 | 45 | 0.9 (0.6-1.5) |  |  |  |
| Single | 154 | 35 | 1 |  |  |  |
| Occupation |  |  |  |  |  |  |
| Employed | 122 | 40 | 1.0 (0.6-1.6) | 0.928 |  |  |
| Unemployed | 159 | 51 | 1 |  |  |  |
| Sex of child |  |  |  | 0.206 |  |  |
| Male | 80 | 117 | 0.8 (0.3-1.2) |  |  |  |
| Female | 82 | 92 | 1 |  |  |  |
| Age of child |  |  |  |  |  |  |
| ≤11 months | 138 | 24 | 0.4 (0.2-0.9) | 0.001* | 1.0 (0.9-1.1) | 0.921 |
| >11months | 195 | 15 | 1 |  | 1 |  |
| Child often has malaria |  |  |  |  |  | 0.023* |
| Yes | 140 | 20 | 3.1 (1.7-5.7) | <0.0001* | 2.0 (1.1-3.8) |  |
| No | 145 | 64 |  |  |  |  |
| Where did you seek care when your child was sick | | |  | 0.689 |  |  |
| Health facility | 117 | 25 | 0.9 (0.6-2.2) |  |  |  |
| Other | 117 | 25 |  |  |  |  |
| Access to NMCP information |  |  |  | 0.207 |  |  |
| Yes | 131 | 30 | 0.7 (0.4-1.3) |  |  |  |
| No | 181 | 29 | 1 |  |  |  |
| Malaria knowledge |  |  |  | 0.276 |  |  |
| Good | 149 | 12 | 0.6 (0.2-1.6) |  |  |  |
| Poor | 200 | 10 | 1 |  |  |  |
| Well-closed house |  |  |  |  |  |  |
| Yes | 132 | 30 | 1.0 (0.6-1.7) | 0.916 |  |  |
| No | 172 | 38 | 1 |  |  |  |
| Well screened windows |  |  |  |  |  |  |
| Yes | 132 | 30 | 1.0 (0.6-1.7) | 0.916 |  |  |
| No | 172 | 30 | 1 |  |  |  |
| Distance between residence to mine sites (≤25 m) | |  |  |  |  |  |
| Yes | 143 | 19 | 4.6 (2.6-8.5) | <0.001* | 1.4 (0.8-2.4) | 0.719 |
| No | 130 | 80 | 1 |  | 1 |  |
| Distance from residence to stagnant water (≤25 m) | | |  |  |  |  |
| Yes | 108 | 54 | 2.8 (1.7-4.2) | <0.001* | 2.9 (1.5-5.8) | 0.002 |
| No | 90 | 120 | 1 |  | 1 |  |
